# A humanized mouse model identifies key amino acids for low immunogenicity of H7N9 vaccines

**DOI:** 10.1038/s41598-017-01372-5

**Published:** 2017-04-28

**Authors:** Yamato Wada, Arnone Nithichanon, Eri Nobusawa, Leonard Moise, William D. Martin, Norio Yamamoto, Kazutaka Terahara, Haruhisa Hagiwara, Takato Odagiri, Masato Tashiro, Ganjana Lertmemongkolchai, Haruko Takeyama, Anne S. De Groot, Manabu Ato, Yoshimasa Takahashi

**Affiliations:** 1 Department of Immunology, National Institute of Infectious Diseases, Tokyo, 162-8640 Japan; 2 Department of Life Science and Medical Bioscience, Waseda University, Tokyo, 162-8480 Japan; 3 Center for Research and Development of Medical Diagnostic Laboratories (CMDL), Faculty of Associated Medical Sciences, Khon Kaen University, 40002 Khon Kaen, Thailand; 4 Influenza Virus Research Center, National Institute of Infectious Diseases, Tokyo, 208-0011 Japan; 5 Institute for Immunology and Informatics, University of Rhode Island, Providence, RI USA; 6 EpiVax Inc, Providence, RI USA; 7 Department of Infection Control Science, Graduate School of Medicine, Juntendo University, Tokyo, 113-8421 Japan; 8 Hagiwara Clinic, Tokyo, 173-0016 Japan

**Keywords:** Viral infection, Protein vaccines

## Abstract

Influenza vaccines of H7N9 subtype are consistently less immunogenic in humans than vaccines developed for other subtypes. Although prior immunoinformatic analysis identified T-cell epitopes in H7 hemagglutinin (HA) which potentially enhance regulatory T cell response due to conservation with the human genome, the links between the T-cell epitopes and low immunogenicity of H7 HA remains unknown due to the lack of animal models reproducing the response observed in humans. Here, we utilized a humanized mouse model to recapitulate the low immunogenicity of H7 HA. Our analysis demonstrated that modification of a single H7 epitope by changing 3 amino acids so that it is homologous with a known H3 immunogenic epitope sequence significantly improved the immunogenicity of the H7 HA in the humanized mouse model, leading to a greater than 4-fold increase in HA-binding IgG responses. Thus, we provide experimental evidence for the important contribution of this H7-specific T cell epitope in determining the immunogenicity of an influenza vaccine. Furthermore, this study delineates strategies that can be used for screening and selecting vaccine strains using immunoinformatics tools and a humanized mouse model.

## Introduction

Influenza virus infections continuously cause severe morbidity and mortality, contributing to 250,000–500,000 annual deaths worldwide^1^. Vaccines that evoke memory antibody responses with virus-neutralizing activity are the best prophylaxis against influenza virus infection^2^. Unfortunately for vaccine developers, influenza A virus has multiple subtypes related to the sequence variation of two key envelope antigens, the hemagglutinin (HA) and neuraminidase (NA) proteins. Seasonal strains of the H1N1 and H3N2 subtypes persist in human populations by constantly acquiring mutations (antigenic drift) which enable them to escape from protective HA-binding antibodies^3, 4^. As a result, almost all adults have been exposed to seasonal influenza viruses and possess pre-existing immunity, primarily consisting of memory B cells, long-lived plasma cells, CD4^+^ memory T cells, and CD8^+^ memory T cells.

Due to the existence of immunological memory, seasonal influenza vaccines can be given as a single dose annually boosting humoral memory responses^5^. Conventional formulations of the seasonal vaccines (split-virion type) drive the differentiation of affinity-matured memory B cells into plasma cells that produce high-affinity antibodies with the help of CD4^+^ memory T cells^6^. In contrast, newly emerging viruses were believed not to boost humoral memory responses as effectively as seasonal vaccines due to the absence of pre-existing memory B and/or CD4^+^ T cells. However, this classical view was recently revised after the analysis of human immune responses against pandemic H1N1 (pH1N1) vaccines; protective immune responses were observed, despite the lack of prior exposure^7–10^.

Split-virion pH1N1 vaccines containing 15 μg HA unexpectedly elicited hemagglutination inhibition antibody titers >1:40 in 94.3–98% of healthy adults^7–10^ with an estimated 87.3% effectiveness^11^. These clinical data demonstrated that pH1N1 vaccines were able to drive protective immune responses after a single dose of unadjuvanted vaccine, despite the lack of previous exposure to homologous virus infection. One explanation for this observation was that a large number of T-cell epitopes were found to be conserved between pH1N1 and seasonal H1/H3 HAs, and that exposure to pH1N1 effectively boosted pre-existing CD4^+^ memory T cells that had been primed by exposure or vaccination with seasonal influenza strains^12–15^. Moreover, the numbers of CD4^+^ memory T cells were found to correlate well with the magnitude of the antibody responses following pH1N1 vaccination^16^ and infection^17^. Based on these observations, it is generally accepted that pH1N1 vaccination leads to the expansion of the pre-existing CD4^+^ memory T cells to cross-conserved HA epitopes, supporting the rapid production of HA-binding antibodies.

Similarly, the efficacies of vaccines against other newly emerging influenza subtypes are expected to be related to the presence of cross-reactive CD4^+^ memory T cells that are generated by seasonal viruses and/or vaccines. For example, avian influenza H5N1 and H7N9 viruses share a number of T cell epitopes with seasonal viruses^18–20^ as do pH1N1 viruses^12–15^. However, vaccines against both H5N1 and H7N9 are poorly immunogenic, resulting in only 10–26% (H5N1) and 1% (H7N9) seroconversion rates, respectively, after a single vaccination with non-adjuvanted formulations^21–23^. Clinical trials of H7N9 vaccines have required the use of adjuvant to increase the seroconversion rates to an acceptable level. Even when two doses of H7N9 vaccine were administered with adjuvant to generate new memory T cells to the novel virus, only 47% of subjects sero-converted in a recent Phase II clinical trial^23^. The development of neutralizing antibodies to H7N9 is also delayed in H7N9-infected humans when compared to the typical immune responses to other influenza virus infections and IgG avidity to H7N9 HA is significantly lower^24^. In clinical trials of other H7 subtypes, an attenuated H7N1 vaccine elicited low HI titers^25^, and an inactivated subunit H7N7 vaccine was poorly immunogenic^26^.

These observations suggest that additional antigenic determinants other than cross-reactive T-cell epitopes may regulate vaccine-induced memory responses. In support of this concept, the previous immunoinformatics analysis identified H7-specific T-cell epitopes that were highly conserved with the human genome (human-like), an *in silico* signature that could potentiate regulatory T cell (Treg) responses^27^. H7 peptides comprising the human-like epitopes expanded CD25^high^FoxP3^+^CD4^+^ T cells in human PBMC cultures *in vitro*, and that co-incubation of the H7 peptides could suppress the effector T-cell responses induced by other H7-immunogenic peptides^28^. However, *in vivo* evidence supporting links between H7-specific T-cell epitopes and the low immunogenicity of H7 HA has been limited to date.

Here, we established a humanized mouse model to recapitulate serological memory responses following influenza vaccination. Our data demonstrated that the substitution of a single H7-specific T cell epitope in the H7 HA protein with an immunogenic H3 consensus sequence significantly improved the immunogenicity of the vaccine in a humanized mouse model. These results provide strong experimental evidence of the contribution of this H7-specific epitope to the lack of immunogenicity of H7N9 vaccines, as was previously proposed^28^.

## Results

### H7N9 and H3N2 vaccines show comparable immunogenicity in naïve mice

**Figure 1:**
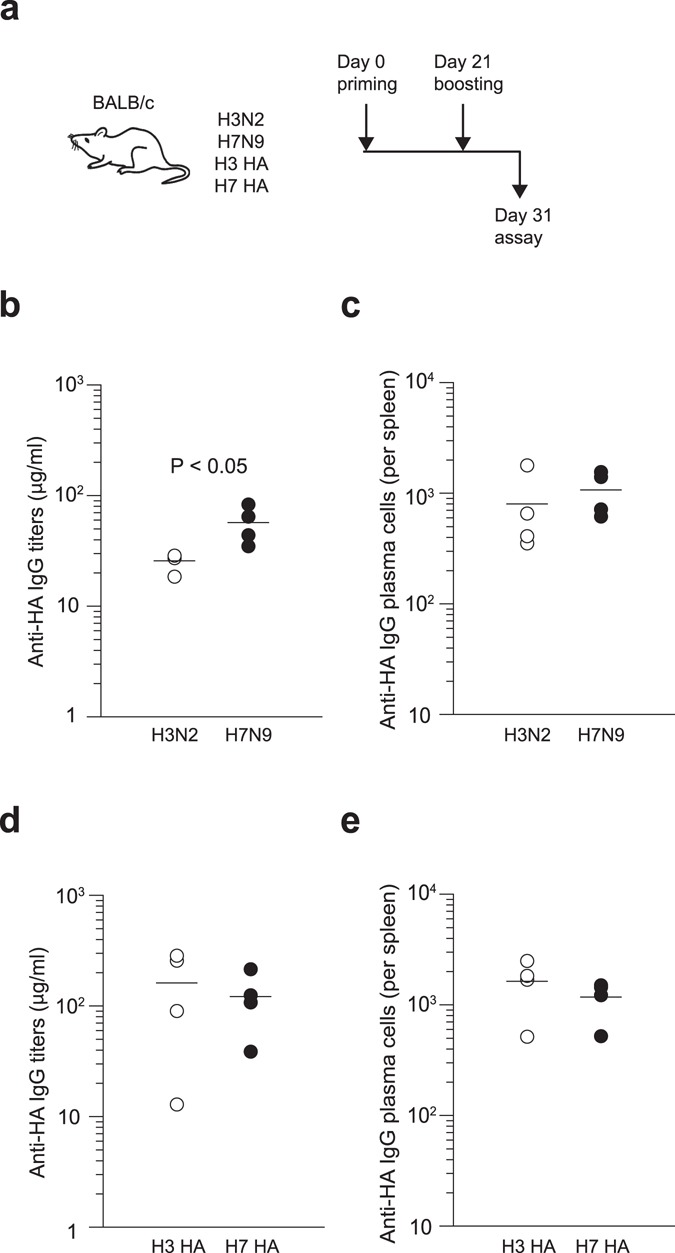
Memory antibody responses elicited by both H7N9 and H3N2 vaccines in naïve mice. As shown in experimental scheme (**a**), BALB/c mice were immunized intraperitoneally twice at a 3-week interval with 10 μg of inactivated whole-virion vaccines (**b**,**c**) or with recombinant HA proteins (**d**,**e**) derived from H7N9 and H3N2 strains. Ten days after boost immunization, sera and splenocytes were collected and analyzed for anti-HA IgG titers using ELISA (**b**,**d**) and anti-HA IgG plasma cells using ELISPOT assay (**c**,**e**). Each circle represents the result from an individual mouse. The difference between H3N2 and H7N9 vaccines was statistically evaluated by a Mann-Whitney test. The data shown are representative of two independent experiments.

There are two phylogenic groups in HA of influenza A viruses; group 1 (*e*.*g*. H1 and H5 subtypes) and group 2 (*e*.*g*. H3 and H7 subtypes). First, we compared the immunogenicity of inactivated whole-virion vaccines between phylogenetically related H3N2 and H7N9 subtypes in the same group 2 using inbred BALB/c mice. Each group of mice was primed and boosted intraperitoneally at 3-week intervals with a non-adjuvanted formulation of the same vaccine. Immune sera were collected at day 10 after boost as shown in Fig. 1a. V15–5 monoclonal antibody, which binds to both H3 and H7 subtypes with similar binding affinity, was used as the standard antibody for quantification of antibody titers, thus allowing for the direct comparison of antibody titers to both subtypes in parallel. At day 10 after boosting, the H7N9 whole-virion vaccines elicited anti-HA IgG titers at slightly but significantly higher levels than the H3N2 whole-virion vaccines (Fig. 1b), although the numbers of anti-HA IgG plasma cells were comparable (Fig. 1c).

Whole-virion vaccines contain other non-HA viral proteins or specific viral particle structures which can potentially influence their immunogenicity. Therefore, we prepared baculovirus-produced HA protein, which is a vaccine formulation approved for human use. The data obtained with the HA protein showed that H3 and H7 HAs were similarly immunogenic to elicit comparable levels of anti-HA IgG titers and plasma cells in inbred BALB/c mice (Fig. 1d and e). The implication of these studies is that standard mouse models could not replicate the low immunogenicity of H7 HA observed in clinical studies, because BALB/c mice were naïve to both H3 and H7 HAs, while human populations were naïve to H7 HA but were immune to H3 HA. However, it remains possible that H7-specific T-cell epitopes identified by the previous analysis^28^ modulate the immunogenicity of H7 HA at some extent and this question is better addressed in the models that mimic HLA-restricted immune responses in humans.

### Humanized mouse models can recapitulate memory antibody responses in humans after influenza vaccination

**Figure 2:**
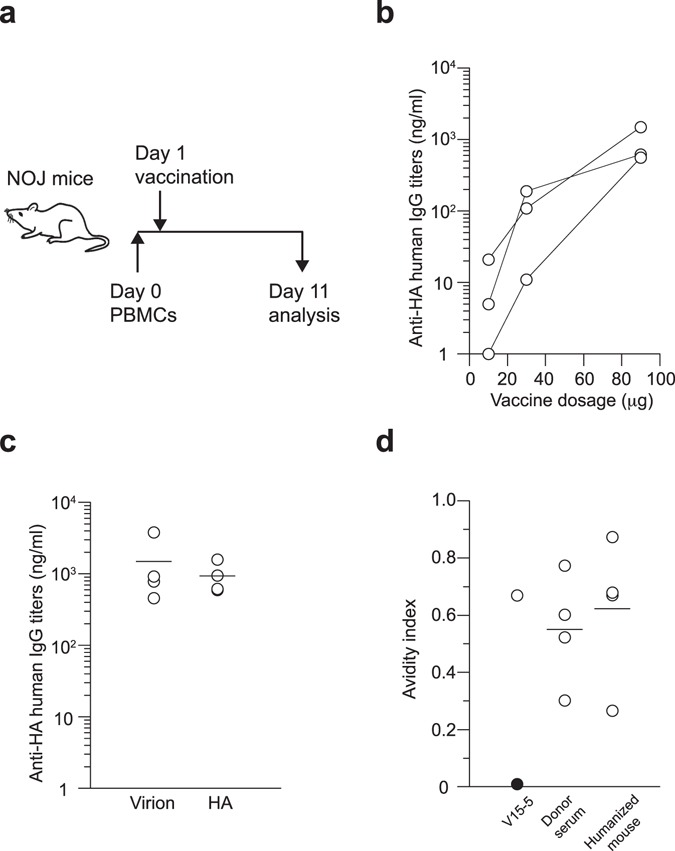
Humanized mouse models to recapitulate memory antibody responses after influenza vaccination. (**a**) NOJ mice were reconstituted with human PBMCs and 24 h later were boosted intravenously with influenza vaccines. At day 10 post-vaccination, sera were collected and analyzed for anti-HA IgG titers by ELISA as shown in a. (**b**) NOJ mice were boosted with different doses of H3N2 whole-virion vaccines and the anti-HA IgG titers in serum samples were estimated by ELISA. The data from three donors are presented. (**c**) NOJ mice were boosted with 90 μg of either whole-virion or recombinant HA vaccines derived from the H3N2 subtype. Anti-HA IgG titers in serum samples were estimated by ELISA. Each circle represents the result from an individual mouse. The data shown are representative of two independent experiments. (**d**) Avidity index of anti-HA IgG antibodies in the sera of the humanized mice and vaccinated donors were plotted. The avidity indices of mutated (open) or germline-reverted (filled) V15–5 monoclonal antibodies were also plotted. Each circle represents the result for an individual mouse.

We therefore decided to determine whether a humanized mouse model could be used to reproduce vaccine-boosted memory responses in humans which are crucially controlled by HLA-restricted interactions with pre-existing memory T and B cells. The NOD/SCID/Jak3^−/−^ (NOJ) mouse strain has defective T and B cell development through a SCID mutation as well as impaired NK cell development due to the lack of IL-2R-mediated Jak3 signaling. This mouse strain has been successfully used as a recipient for transplanted human cells^29, 30^. As expected, we observed that NOJ mice transplanted with human peripheral blood mononuclear cells (PBMCs) produced human anti-HA IgG antibodies after vaccination with several doses (10, 30, and 90 μg per mouse) of H3N2 whole-virion vaccines (Fig. 2a and b). To reduce the possible donor-to-donor variation, we maximized the antibody response by using 90 μg per mouse as the vaccination dose, even though this dose is higher than the dose that is usually applied in standard mouse models. Similar experiments were performed using a recombinant H3 HA protein resulting in recall responses comparable to those obtained with whole-virion vaccines (Fig. 2c). To further confirm the contribution of recall responses on elicited HA-binding antibodies, we examined antibody avidity indices by using a modified ELISA to dissociate plate-bound, low-affinity antibodies selectively via treatment with 7 M urea (Fig. 2d)^31^. Mutated or germline-reverted forms of V15–5 monoclonal antibodies were assayed in parallel as controls. We also determined the avidity indices of serum antibodies from vaccinated donors as positive controls. The data clearly demonstrate that humanized mice developed antibody responses with equivalent avidity indices to those from serum of vaccinated donors and affinity-matured monoclonal antibody. Therefore, our results demonstrate that this humanized mouse model can reproduce vaccine-boosted memory responses and the development of high affinity antibody responses, and that it can be used to evaluate the human immunogenicity of influenza vaccines *in vivo*.

### H7 HA is poorly immunogenic in a humanized mouse model

**Figure 3:**
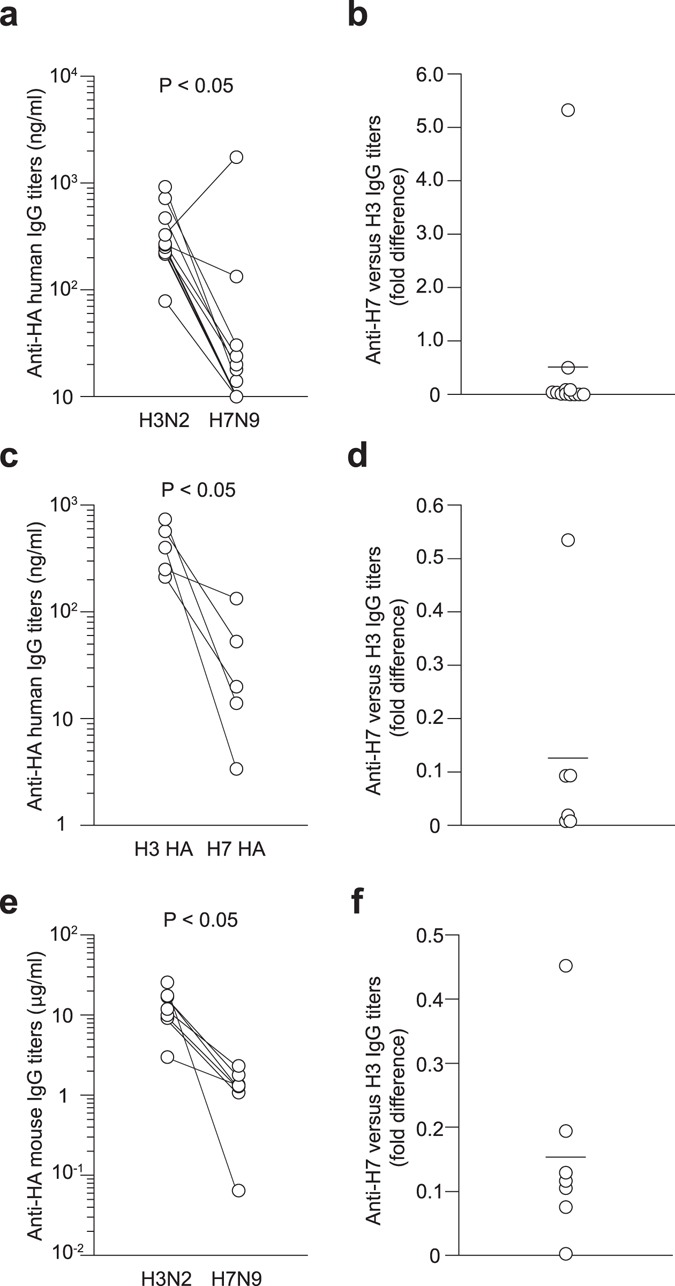
Low immunogenicity of H7N9 vaccines revealed in a humanized mouse model. Pair of humanized mice were prepared from the same donors, boosted intravenously with 90 μg of either whole-virion (**a**,**b**) or recombinant HA (**c**,**d**) vaccines and assayed for human anti-HA IgG titers (**a**,**c**) at day 10 post-vaccination using V15–5 monoclonal antibody as standard. Fold differences in anti-H7 versus H3 IgG titers are plotted in the right panel (**b**,**d**). The difference in antibody responses between H3N2 and H7N9 vaccinated groups was statistically evaluated by a Wilcoxon matched-pairs signed rank test. Each circle represents the result from an individual mouse. (**e**,**f**) NOJ mice were reconstituted with H3N2-primed mouse splenocytes and then boosted with 90 μg of whole-virion vaccines. Mouse anti-HA IgG titers were assessed at day 10 post-vaccination. The difference between H3N2 and H7N9 vaccinated groups was statistically evaluated by a Wilcoxon matched-pairs signed rank test.

Having demonstrated that the humanized mouse model could reproduce human memory responses against influenza vaccines, we then examined whether immunization of the mice with H7N9 vaccines would recapitulate previous observations that the vaccines were poorly immunogenic in humans, as demonstrated repeatedly in clinical trials^23, 32, 33^. Pairs of humanized mice were prepared using PBMCs from the same donor. One mouse from each pair received H3N2 whole-virion vaccine (control group) and the second mouse received H7N9 whole-virion vaccine (experimental group). Side-by-side analysis of each pair of humanized mice from the same donor increased the sensitivity of the assay by reducing the negative impact of donor-to-donor variability caused by different genetic backgrounds and immune histories. Indeed, there was considerable intragroup variability in the antibody response elicited by both control (H3N2) and experimental (H7N9) groups (Fig. 3a and b). Despite this variability, significantly lower antibody responses were observed in the H7N9-vaccinated groups (Fig. 3a and b) compared to those in the H3N2-vaccinated control group, with only two mice showing similar or higher responses against the H7N9 vaccines. We also performed the same study using recombinant H3 and H7 HA vaccines to exclude the possibility that other internal viral proteins present in the whole-virion vaccines could influence the HA-binding antibody response due to the utility of T cells responding to non-HA antigens^34^. The recombinant HA vaccines completely reproduced the results observed with whole-virion vaccines, in that H7 HA vaccination was only able to induce weak HA-binding antibody recall responses (Fig. 3c and d), as compared to H3 HA vaccination in the mice transplanted with PBMCs from the same donor. From these results, we concluded that this humanized mouse model can recapitulate the low levels of HLA-restricted antibody response elicited by H7 HA, as observed in clinical trials.

As previously noted, one explanation for the poor immunogenicity of H7 HA in human PBMC-transplanted NOJ mice is lack of prior H7N9 exposure in humans, which contrasts with greater levels of antibodies against H3 HA due to the pre-existing immunity. To assess the contribution of prior exposure to H3 HA, NOJ mice were transplanted with murine lymphocytes from H3N2-exposed mice instead of human PBMCs. Consistent with the data from human PBMC-transplanted NOJ mice, H7 HA vaccination of NOJ mice transplanted with H3N2-primed mouse lymphocytes elicited low anti-H7 HA IgG responses (Fig. 3e,f). Given the comparable immunogenicity of H3 and H7 HA in naïve mice (Fig. 1), these results suggest that the lack of immune history to H7 HA substantially contributes to low antibody responses to H7 HA vaccines in NOJ mice transplanted with H3N2-primed lymphocytes.

### Mutation of H7-specific T-cell epitope improves the immunogenicity of H7 HA in a humanized mouse model

**Figure 4:**
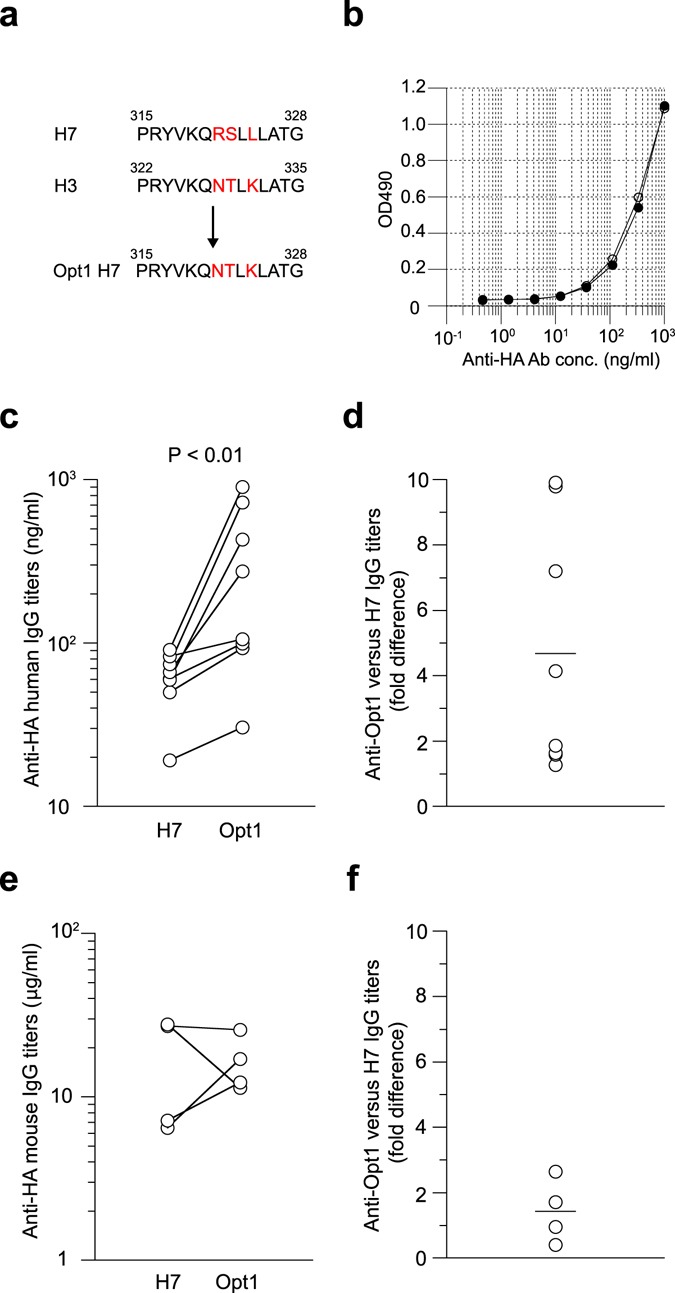
Enhanced immunogenicity of Opt1 H7 HA independently of B-cell epitopes. (**a**) H7 T-cell epitope (315–328) and H3 epitope (322–335) in the corresponding region are presented. Opt1 H7 HA was designed to reduce the levels of molecular mimicry in the H7 T-cell epitope by substitution with an immunogenic H3 epitope. Key amino acid residues that differ between H7 and H3 are highlighted in red. (**b**) Wild-type H7 HA (open) and Opt1 H7 HA (filled) were compared for recognition by polyclonal H7 HA-binding IgG antibodies present in human serum using ELISA. (**c**) Pair of humanized mice were prepared from the same donors, boosted intravenously with 90 μg of H7 or Opt1 H7 HA vaccines, and assessed for human anti-HA IgG titers at day 10 post-vaccination using V15–5 monoclonal antibody as standard. The difference between H7 and Opt1 H7 HA vaccinated groups was statistically evaluated by a Wilcoxon matched-pairs signed rank test. Each circle represents the result from individual mouse. (**d**) Fold differences in anti-Opt1 versus H7 HA IgG titers are plotted. (**e**) NOJ mice were reconstituted with H3N2-primed mouse splenocytes and then boosted with 90 μg of H7 or Opt1 H7 HA vaccine. Thereafter, mouse anti-HA IgG titers were assessed at day 10 post-vaccination. The difference between H7 and Opt1 H7 HA vaccinated groups was statistically evaluated by a Wilcoxon matched-pairs signed rank test. Each circle represents the result from individual mouse. (**f**) Fold differences in anti-Opt1 versus H7 HA IgG titers are plotted.

Previous immunoinformatics analysis identified H7-specific T-cell epitopes that may potentially enhance Treg responses in HLA-restricted manner, which could also affect the magnitude of antibody responses elicited by vaccines^28^. One such T cell epitope corresponding to amino acid residues 315 to 328 in the H7 HA (Fig. 4a) was previously shown to expand FoxP3^+^ Treg cells in PBMCs after *in vitro* stimulation, which in turn, correlated with the partial suppression of effector T-cell responses against other H7N9 peptides by co-incubation with this peptide^28^. In order to assess a possible contribution of the H7-specific T-cell epitopes on the observed low immunogenicity of H7 HA, we designed and generated a H7 HA mutant protein (designated as Opt1 H7 HA) containing only 3 amino acid replacements, which converted the H7 epitope into the sequence of an immunogenic H3 epitope found in the same region of the H3 HA protein (Fig. 4a).

The Opt1 H7 HA protein was produced in a baculovirus expression system (same as for wild-type H7 HA), and then assayed for binding against polyclonal H7 HA-binding IgG antibodies present in the serum of healthy volunteers to evaluate the effect of the amino acid substitutions on HA antigenicity (Fig. 4b). Opt1 H7 HA and wild-type H7 HA protein was equally well recognized by polyclonal H7 HA-binding IgG antibodies in human sera, indicating that B-cell epitopes remained intact in Opt1 H7 HA. We then examined T-cell related effects that could be caused by the three amino acid replacements introduced in Opt1 H7 HA. As demonstrated in Fig. 3, pairs of humanized mice were prepared using PBMCs from the same donor. One of the mice from each pair was immunized with the wild-type H7 HA vaccine (control group) and the other mouse with Opt1 H7 HA vaccine (experimental group). We observed that Opt1 H7 HA vaccines induced significantly higher levels of anti-HA IgG responses, resulting in >4-fold increases in antibody titers compared to wild-type H7 HA vaccines (Fig. 4c and d). Intriguingly, half of the humanized mice responded better to Opt1 H7 HA, while another half responded to Opt1 H7 HA in a range similar to wild-type H7 HA. The high and low responders may be explained by HLA haplotypes or differential status of memory T cell responses/Treg responses in donors. Further investigations are required to address these possibilities.

To determine whether this result was specific to HLA-restricted immune responses in humans, the same set of vaccines was administrated into NOJ mice that had received H3N2-primed murine lymphocytes as previously described (Fig. 3e). Despite prior exposure to H3N2, we did not observe any significant difference in boosting ability between wild-type H7 HA and Opt1 H7 HA (Fig. 4e and f), suggesting that the difference observed in the human PBMC-transplanted mice were related to HLA-restricted (and not mouse MHC-restricted) immune responses rather than to previous exposure to H3 HA. Collectively, these results strongly support the notion that H7 HA is poorly immunogenic, at least partly, due to the presence of HLA-restricted human-like T cell epitopes and/or the absence of immunogenic T-cell epitope, as was predicted by immunoinformatics analysis^28^.

## Discussion

Unexpected clinical findings from non-adjuvanted pH1N1 vaccines have contributed to a revision of classical views of memory antibody responses to influenza vaccines. Cross-reactive CD4^+^ memory T cells have been identified as key determinants of immunogenicity for pH1N1 vaccines and viruses^16, 17^. It has been more difficult to explain the poor immunogenicity of H5N1 and H7N9 vaccines, since these strains do share cross-conserved T-cell epitopes with seasonal influenza strains to some extent^18–20^. The results presented in this paper provide experimental evidence that H7-specific T cell epitopes significantly affect the immunogenicity of H7 influenza vaccines and suggest two compatible possibilities for this observation. One is molecular mimicry that negatively impacts on the immunogenicity of H7 HA through immune tolerance and the other is a lack of cross-conserved effector T-cell epitopes that enhances the immunogenicity. The previous study supports the former possibility, as the epitope sequence targeted for modification expanded CD25^high^FoxP3^+^CD4^+^ T cells in human PBMC cultures *in vitro*, and co-incubation of the epitope could suppress effector T-cell responses stimulated by other H7 immunogenic peptides^28^. The relative contribution of seasonal influenza epitopes newly introduced into H7 HA to improve antibody responses remains an important question.

This paper also demonstrates the usefulness of NOJ mice reconstituted with human PBMCs for evaluating the immunogenicity of influenza vaccines. A similar humanized mouse system using SCID/SCID mice as recipient has been successfully applied to the analysis of humoral recall responses to pneumococcal vaccines^35^; however, to our knowledge, this is the first report focusing *in vivo* recapitulation of the comparative immunogenicity of influenza vaccines observed in humans. One caveat of this mouse system is that anti-HA IgG antibody titers in human cell-transplanted NOJ mice (0.35 μg/ml) were lower than those in mouse cell-transplanted NOJ mice (14 μg/ml) (Fig. 3). Possibly due to the low recall responses, human T-cell responses in the same humanized mice were below the detection limit (data not shown), hampering further analysis of T cell responses that are responsible for low immunogenicity of H7 HA. Because Opt1 H7 HA showed comparable immunogenicity to wild-type H7 HA in murine cell-transplanted NOJ mice (Fig. 4e and f), we surmise the increased antibody responses observed in human cell-transplanted mice may be attributed to species-specific events such as differential specificities, frequencies, and functions of human CD4^+^ T cells. In this context, *in vitro* human PBMC cultures in a prior study showed that wild-type H7-specific epitope (315–328) expanded CD25^high^FoxP3^+^CD4^+^ Treg cells and reduced IFN-γ secretion from T cells stimulated with other H7 epitopes^28^. Thus, substitution of the H7-specific epitope with a highly conserved H3 epitope under HLA restriction could avoid Treg induction by the wild-type H7 and boost CD4 T cell responses in human cell-transplanted mice.

Cognate interaction of memory T and B cells mediated by HLA-restricted T-cell epitope is one of the most important factors regulating the magnitude and quality of recalled memory responses^36^. At least one subset of memory T and B cells, called central memory, circulates in the blood, making it possible to recapitulate the cognate interactions between human memory T and B cells in mice using a humanized mouse model system^37^. Indeed, we found that the affinity maturation of elicited antibody responses in humanized mice was similar to those in vaccinated donors, supporting the idea that the reproduced responses are mainly composed of recall responses rather than primary responses. Thus, the humanized NOJ mouse model appears to be useful for vaccine studies, since it can mimic the human recall responses to protein-based influenza vaccines, making it a suitable model for use in preclinical trials. While it is conceivable that innate cells and stromal cells also play roles in enhancing recall responses^38^, the effects of these cells are largely modulated by non-protein composition of vaccines, especially the ligands of pattern-recognition receptors, which are not included in HA protein-based vaccines.

The study may also introduce the concept of host mimicry into influenza viruses. Many chronically infecting viruses, such as Epstein-Barr virus, herpes simplex virus, human immunodeficiency virus, hepatitis C virus, and cyotomegaloviruses, may avoid or attenuate effector T-cell responses by mimicking to a self-antigen, thereby limiting effector T-cell responses or causing the expansion and activation of Treg cells^39, 40^. A comprehensive analysis of the full genomes of these human pathogens demonstrated that these viruses contained fewer T-cell epitopes and exhibited higher cross-conservation with autologous antigens than did acutely infecting Ebola and Marburg viruses^41^. Furthermore, the immunosuppressive effects of human-like T cell epitopes have been experimentally confirmed for hepatitis C virus (HCV); *in vitro* stimulation of PBMCs by self-mimicking HCV peptides expanded Treg cells^42^, as was also the case for H7N9 epitopes. Thus, it is tempting to speculate that human-like epitopes found in H7 HA is an explanation for reduced H7N9 vaccine efficacy in humans and humanized mice.

While the prevalence of human-like epitopes in human commensals can be interpreted as a mean of viral escape, the presence of human-like epitopes in an avian-origin influenza virus is more difficult to explain. To date, human-to-human transmission of H7N9 is quite rare, as the virus is not fully adapted to humans^43, 44^. One explanation for the presence of human-like epitopes could be that these epitopes contribute to the success of H7N9 propagation in their usual hosts. Models for avian MHC are not yet available, thus it is difficult to determine whether epitopes like the one validated here contributes to immune escape by H7N9 in chickens or other birds. Future serological studies in humans, other mammals, and poultry, as well as additional investigation into the potential contribution of Treg responses in non-human hosts, may shed light on the origin of H7-specific T-cell epitopes that affect the immunogenicity of influenza vaccines.

## Methods

### Immunoinformatic design of Opt1 H7 HA

The amino acid sequence of the influenza A/Shanghai/2/2013 HA (H7) was obtained from GISAID (www.gisaid.org). The retrieved sequence was parsed into overlapping 9-mer frames and screened for the presence of predicted class II restricted T cell epitopes using the EpiMatrix system^45^. ClustiMer, an algorithm used to identify short amino acid sequences containing multiple HLA binding motifs, was used to identify potential T cell epitope clusters contained within the H7 HA sequence^46^. Finally, the JanusMatrix algorithm was used to identify putative T cell epitopes contained within H7 HA which share TCR contacts with putative epitopes present in the human genome^47^.

### Vaccine preparation

NIBRG-268 (A/Anhui/1/2013, H7N9) virus was provided by the National Institute for Biological Standards and Controls. IVR-165 (A/Victoria/361/2011, H3N2) virus was provided by the Commonwealth Serum Laboratories. Both viruses were propagated on embryonated chicken eggs and purified through a 10–50% sucrose gradient as previously described^48^. For inactivation, purified viruses were treated with 0.1% formalin at 4 °C for a week and used as inactivated whole-virion vaccines. Recombinant HA protein was produced in a baculovirus expression system (Clontech) as previously described^49^.

### Mice and vaccination

BALB/c mice were purchased from Japan SLC and NOD/SCID/Jak3^−/−^ mice were kindly provided by Dr. S. Okada (Kumamoto University) and maintained in our institute. All mice were maintained under SPF condition and used at 8–15 weeks of age. BALB/c mice were intraperitoneally immunized with whole-virion or HA vaccines (10 μg per mouse) and boosted by the same dose of vaccines. At day 10 after boosting, sera were recovered from tail veins and subjected to ELISA for measuring anti-HA IgG antibody titers. Spleen cells were also recovered after boosting and then evaluated by ELISPOT assay for enumerating anti-HA IgG plasma cells. In some experiments, BALB/c mice were intranasally infected with H3N2 (X31) virus as previously described^49^ and were used as the source of H3N2-primed splenocytes at more than 2 months after infection.

The humanized mouse model was developed as follows; heparinized peripheral blood was obtained from healthy donors with written informed consent and PBMCs were freshly isolated by density centrifugation using Ficoll-Hypaque (GE Healthcare). Two mice were intravenously transplanted with 2–5 × 10^7^ PBMCs from each donor. Twenty-four hours after transplantation of PBMCs, the mice were intravenously vaccinated and the serum and spleens were collected at day 10. In some experiments, the splenocytes from H3N2-primed mice were used for transplantation. All animal experiments were approved by the animal experimental committees of the National Institute of Infectious Diseases, Japan, and performed in accordance with the guidelines of the Institutional Animal Care and Use Committee. The studies using human samples were approved by Institutional Ethics Committee of Human Experimentation and performed in accordance with the Ethical Guidelines for Medical and Health Research Involving Human Subjects in Japan.

### ELISA and ELISPOT

For the detection of anti-HA plasma cells with ELISPOT assay, nitrocellulose membranes were coated with 10 μg/mL recombinant HA, and cells were incubated on the membranes for 3 h at 37 °C. After the cells were washed off, the membranes were incubated with anti-mouse IgG–HRP (Southern Biotech). HRP activities were visualized as previously described^49^. For the detection of anti-HA antibody titers, ELISA was performed with anti-mouse IgG-HRP, or anti-human IgG-HRP as previously described^50^. The avidity index was calculated by dividing anti-HA IgG antibody titers resistant to 7 M urea treatment (15 min) by total anti-HA IgG antibody titers. For standard antibodies, we used IgG1 monoclonal antibody (V15–5) with comparable binding to H3 and H7 HA.

### Statistical analysis

Statistical significance was determined using a two-tailed, nonparametric Mann-Whitney test or Wilcoxon matched-pairs signed rank test. P-values less than 0.05 were considered significant.

## References

[1] *World Health Organization Influenza* (*Seasonal*), http://www.who.int/mediacentre/factsheets/fs211/en/ (2014).

[2] Cox NJ, Subbarao K (1999). Influenza. Lancet.

[3] Wiley DC, Wilson IA, Skehel JJ (1981). Structural identification of the antibody-binding sites of Hong Kong influenza haemagglutinin and their involvement in antigenic variation. Nature.

[4] Skehel JJ, Wiley DC (2000). Receptor binding and membrane fusion in virus entry: the influenza hemagglutinin. Annu Rev Biochem.

[5] Fiore AE, Bridges CB, Cox NJ (2009). Seasonal influenza vaccines. Curr Top Microbiol Immunol.

[6] Onodera T (2016). Whole-virion influenza vaccine recalls an early burst of high-affinity memory B cell response through TLR signaling. J Immunol.

[7] Greenberg ME (2009). Response to a monovalent 2009 influenza A (H1N1) vaccine. N Engl J Med.

[8] Liang XF (2010). Safety and immunogenicity of 2009 pandemic influenza A H1N1 vaccines in China: a multicentre, double-blind, randomised, placebo-controlled trial. Lancet.

[9] Plennevaux E, Sheldon E, Blatter M, Reeves-Hoche MK, Denis M (2010). Immune response after a single vaccination against 2009 influenza A H1N1 in USA: a preliminary report of two randomised controlled phase 2 trials. Lancet.

[10] Zhu FC (2009). A novel influenza A (H1N1) vaccine in various age groups. N Engl J Med.

[11] Wu J (2010). Safety and effectiveness of a 2009 H1N1 vaccine in Beijing. N Engl J Med.

[12] Greenbaum JA (2009). Pre-existing immunity against swine-origin H1N1 influenza viruses in the general human population. Proc Natl Acad Sci USA.

[13] Richards KA, Topham D, Chaves FA, Sant AJ (2010). Cutting edge: CD4 T cells generated from encounter with seasonal influenza viruses and vaccines have broad protein specificity and can directly recognize naturally generated epitopes derived from the live pandemic H1N1 virus. J Immunol.

[14] Ge X (2010). Assessment of seasonal influenza A virus-specific CD4 T-cell responses to 2009 pandemic H1N1 swine-origin influenza A virus. J Virol.

[15] Subbramanian RA, Basha S, Shata MT, Brady RC, Bernstein DI (2010). Pandemic and seasonal H1N1 influenza hemagglutinin-specific T cell responses elicited by seasonal influenza vaccination. Vaccine.

[16] Nayak JL (2013). CD4+ T-cell expansion predicts neutralizing antibody responses to monovalent, inactivated 2009 pandemic influenza A(H1N1) virus subtype H1N1 vaccine. J Infect Dis.

[17] Wilkinson TM (2012). Preexisting influenza-specific CD4+ T cells correlate with disease protection against influenza challenge in humans. Nat Med.

[18] Lee LY (2008). Memory T cells established by seasonal human influenza A infection cross-react with avian influenza A (H5N1) in healthy individuals. J Clin Invest.

[19] Roti M (2008). Healthy human subjects have CD4+ T cells directed against H5N1 influenza virus. J Immunol.

[20] Richards KA (2015). Seasonal influenza can poise hosts for CD4 T-cell immunity to H7N9 avian influenza. J Infect Dis.

[21] Bresson J-L (2006). Safety and immunogenicity of an inactivated split-virion influenza A/Vietnam/1194/2004 (H5N1) vaccine: phase I randomised trial. Lancet.

[22] Treanor JJ, Campbell JD, Zangwill KM, Rowe T, Wolff M (2006). Safety and immunogenicity of an inactivated subvirion influenza A (H5N1) vaccine. N Engl J Med.

[23] Mulligan MJ (2014). Serological responses to an avian influenza A/H7N9 vaccine mixed at the point-of-use with MF59 adjuvant: a randomized clinical trial. JAMA.

[24] Guo L (2014). Human antibody responses to avian influenza A(H7N9) virus, 2013. Emerg Infect Dis.

[25] Krammer F (2014). An H7N1 influenza virus vaccine induces broadly reactive antibody responses against H7N9 in humans. Clin Vaccine Immunol.

[26] Couch RB, Patel SM, Wade-Bowers CL, Nino D (2012). A randomized clinical trial of an inactivated avian influenza A (H7N7) vaccine. PLoS One.

[27] De Groot AS (2014). Cross-conservation of T-cell epitopes: now even more relevant to (H7N9) influenza vaccine design. Hum Vaccin Immunother.

[28] Liu R (2015). H7N9 T-cell epitopes that mimic human sequences are less immunogenic and may induce Treg-mediated tolerance. Hum Vaccin Immunother.

[29] Okada S, Harada H, Ito T, Saito T, Suzu S (2008). Early development of human hematopoietic and acquired immune systems in new born NOD/Scid/Jak3null mice intrahepatic engrafted with cord blood-derived CD34+ cells. Int J Hematol.

[30] Satoh M (2010). Evaluation of a recombinant measles virus expressing hepatitis C virus envelope proteins by infection of human PBL-NOD/Scid/Jak3null mouse. Comp Immunol Microbiol Infect Dis.

[31] de Souza VAUF (2004). Use of an immunoglobulin G avidity test to discriminate between primary and secondary dengue virus infections. J Clin Microbiol.

[32] Fries LF, Smith GE, Glenn GM (2013). A recombinant viruslike particle influenza A (H7N9) vaccine. N Engl J Med.

[33] Bart, S. A. *et al*. A cell culture-derived MF59-adjuvanted pandemic A/H7N9 vaccine is immunogenic in adults. *Sci Transl Med* **6**, 234ra255, doi:10.1126/scitranslmed.3008761 (2014). 10.1126/scitranslmed.3008761 24786323

[34] Scherle PA, Gerhard W (1986). Functional analysis of influenza-specific helper T cell clones *in vivo*. T cells specific for internal viral proteins provide cognate help for B cell responses to hemagglutinin. J Exp Med.

[35] Moens L, Wuyts M, Meyts I, De Boeck K, Bossuyt X (2008). Human memory B lymphocyte subsets fulfill distinct roles in the anti-polysaccharide and anti-protein immune response. J Immunol.

[36] Kurosaki T, Aiba Y, Kometani K, Moriyama S, Takahashi Y (2010). Unique properties of memory B cells of different isotypes. Immunol Rev.

[37] Sallusto F, Lenig D, Forster R, Lipp M, Lanzavecchia A (1999). Two subsets of memory T lymphocytes with distinct homing potentials and effector functions. Nature.

[38] Shaw J, Wang YH, Ito T, Arima K, Liu YJ (2010). Plasmacytoid dendritic cells regulate B-cell growth and differentiation via CD70. Blood.

[39] Chevalier MF, Weiss L (2013). The split personality of regulatory T cells in HIV infection. Blood.

[40] Moise L (2016). T cell epitope redundancy: cross-conservation of the TCR face between pathogens and self and its implications for vaccines and autoimmunity. Expert Rev Vaccines.

[41] He L (2014). Integrated assessment of predicted MHC binding and cross-conservation with self reveals patterns of viral camouflage. BMC bioinformatics.

[42] Losikoff PT (2015). HCV epitope, homologous to multiple human protein sequences, induces a regulatory T cell response in infected patients. J Hepatol.

[43] Hu J, Zhu Y, Zhao B, Li J, Liu L, Gu K, Zhang W, Su H, Teng Z, Tang S, Yuan Z, Feng Z, Wu F (2014). Limited human-to-human transmission of avian influenza A(H7N9) virus, Shanghai, China, March to April 2013. Eurosurveillance.

[44] Watanabe T, Watanabe S, Maher EA, Neumann G, Kawaoka Y (2014). Pandemic potential of avian influenza A (H7N9) viruses. Trends Microbiol.

[45] Moise L (2015). iVAX: an integrated toolkit for the selection and optimization of antigens and the design of epitope-driven vaccines. Hum Vaccin Immunother.

[46] Moise L (2013). Universal H1N1 influenza vaccine development: identification of consensus class II hemagglutinin and neuraminidase epitopes derived from strains circulating between 1980 and 2011. Hum Vaccin Immunother.

[47] Moise L (2013). The two-faced T cell epitope: examining the host-microbe interface with JanusMatrix. Hum Vaccin Immunother.

[48] Takahashi Y (2009). Protective immunity afforded by inactivated H5N1 (NIBRG-14) vaccine requires antibodies against both hemagglutinin and neuraminidase in mice. J Infect Dis.

[49] Adachi Y (2015). Distinct germinal center selection at local sites shapes memory B cell response to viral escape. J Exp Med.

[50] Onodera T (2012). Memory B cells in the lung participate in protective humoral immune responses to pulmonary influenza virus reinfection. Proc Natl Acad Sci USA.

