# Publisher Correction: A humanized mouse model identifies key amino acids for low immunogenicity of H7N9 vaccines

**DOI:** 10.1038/s41598-019-50828-3

**Published:** 2019-10-08

**Authors:** Yamato Wada, Arnone Nithichanon, Eri Nobusawa, Leonard Moise, William D. Martin, Norio Yamamoto, Kazutaka Terahara, Haruhisa Hagiwara, Takato Odagiri, Masato Tashiro, Ganjana Lertmemongkolchai, Haruko Takeyama, Anne S. De Groot, Manabu Ato, Yoshimasa Takahashi

**Affiliations:** 1 Department of Immunology, National Institute of Infectious Diseases, Tokyo, 162-8640 Japan; 2 Department of Life Science and Medical Bioscience, Waseda University, Tokyo, 162-8480 Japan; 3 Center for Research and Development of Medical Diagnostic Laboratories (CMDL), Faculty of Associated Medical Sciences, Khon Kaen University, 40002 Khon Kaen, Thailand; 4 Influenza Virus Research Center, National Institute of Infectious Diseases, Tokyo, 208-0011 Japan; 5 Institute for Immunology and Informatics, University of Rhode Island, Providence, RI USA; 6 EpiVax Inc, Providence, RI USA; 7 Department of Infection Control Science, Graduate School of Medicine, Juntendo University, Tokyo, 113-8421 Japan; 8 Hagiwara Clinic, Tokyo, 173-0016 Japan

Correction to: *Scientific Reports* 10.1038/s41598-017-01372-5, published online 28 April 2017

In the original version of this Article, the author Anne S. De Groot was incorrectly indexed.

In addition, the publication date was omitted from the original PDF version of this Article.

These errors have now been corrected in the HTML and PDF versions of the Article.

